# Remote Health Monitoring Systems Based on Bluetooth Low Energy (BLE) Communication Systems

**DOI:** 10.1007/978-3-030-51517-1_4

**Published:** 2020-05-31

**Authors:** Lamia Chaari Fourati, Sana Said

**Affiliations:** 8 Digital Research Centre of Sfax, Sfax, Tunisia; 9 Institut Mines-Télécom, CNRS, Paris, France; 10 Université de Sherbrooke, Sherbrooke, QC Canada; 11 Digital Research Centre of Sfax, Sfax, Tunisia; 12 University of Sfax, Sfax, Tunisia; Digital Research Center of Sfax (CRNS), Laboratory of Technologies and Smart Systems (LT2S), University of Sfax, Sfax, Tunisia

**Keywords:** Remote health monitoring system, Bluetooth Low Energy, Healthcare services

## Abstract

Nowadays, remote healthcare monitoring systems (RHMS) are attracting patients, doctors and caregivers. RHMS reduces the number of unessential hospitalizations by providing the required healthcare services for patients at home. Furthermore, continuous health monitoring using RHMS is a hopeful solution for elderly people suffering from chronic diseases. RHMS is in general three tiers architecture where the first tier uses intelligent wearable sensors to gather physiological signs. The majority of wearable sensors constructors commercialized sensing devices with Bluetooth Low Energy (BLE) communication interfaces, which lead to the development of diverse RHMS deploying BLE communication interfaces for physiological patient data gathering. In this paper, we introduce the basic concepts related to RHMS design and development. Besides that, we focus our investigation on the BLE communication protocol used in the healthcare context and its configuration to sense several physiological data. Also, we highlight the different steps enabling reading sensed data on mobile application.

## Introduction

### General Context

Healthcare is one of the fastest-growing business fields and an important market for most countries and healthcare services are the most needed and consumed service by elderly people in the word. Providing healthcare services for every citizen everywhere becoming possible thanks to remote healthcare monitoring systems (RHMS), which allow long-term management of health conditions, diseases prevention and detection of emergencies. RHMS is based on the deployment of Wireless Body Area Network (WBAN) using wearable and/or implantable sensors in or around the human body. The sensed physiological data are forwarded to collecting node known as data collector or a gateway or a coordinator (smartphone or PDA: Personal Data Assistant) which is connected to the remote server (for data processing and storage) through the internet network (via WiFi) or the cellular networks (4G or 5G) [1]. RHMS could be based on three or four tiers architectures [2]. In this work, we focus our investigation on the first tier. Therefore, different communication technologies were suggested for the data exchange between the body sensors nodes and the coordinator(the first tier). Among the most deployed communication technologies in the first tier, we can highlight the following:

IEEE 802.15.4 [3–6].

IEEE 802.15.6 [7–12].

Bluetooth Low Energy [13–15].

The IEEE 802.15.6 [7] is the dedicated standards for the communication between the sensors and the coordinator. Nowadays, sensing devices with 802.15.6 modules are not available enough for the commercial usage in the market and they are more expensive than wearable sensors with Bluetooth Low Energy (BLE) which are widely commercialized by many sensors constructors such as libelium, mindwave.... For this reason, in this paper, our investigation is related to RHMS using sensors with BLE interfaces.

### Contributions and Paper Organisation

The goal of this article is to study and analyze the most significant efforts related to RHMS integrating BLE communication modules. Accordingly, this paper provides a rich bibliography in the domain that can support future reading in this emerging field and pinpoints the technical issues related to the design and development of the BLE based RHMS. Furthermore, this investigation selects and considers several pioneering studies that can act as a roadmap of this wide-ranging research area. Although there is a lot of research works recently proposed that focused on RHMS, to the best of our knowledge, there is no thorough study that cogitates all BLE based RHMS design and development issues. The main contributions of this paper summarized as follows:

(1) state-of-the-art analysis related to BLE based RHMS for mono and multi physiological data sensing;

(2) comprehensive study and roadmap related to the design and the development of BLE based RHMS;

(3) BLE Processing and computation issues of sensed physiological data.

The rest of this paper is organized as follows: the second section describes the basic architecture of RHMS and analyses some related works about BLE based systems for single or multiple sensors. The third section overviews the basic concepts of communication systems based on BLE. The fourth section describes BLE Processing and computation of sensed physiological data such as ECG, SpO2, EMG, HR, etc...and highlights BLE service characteristics data related to each sensor. Finally, the fifth section concludes the paper and draws our future directions.

## BLE Based RHMS

This section illustrates and explains the general architecture of the RHMS. Then, it studies and analyzes related works focusing on RHMS that integrate BLE communication interfaces between the physiological sensors and the PDA.

### RHMS Basic Architecture

**Fig. 1.**
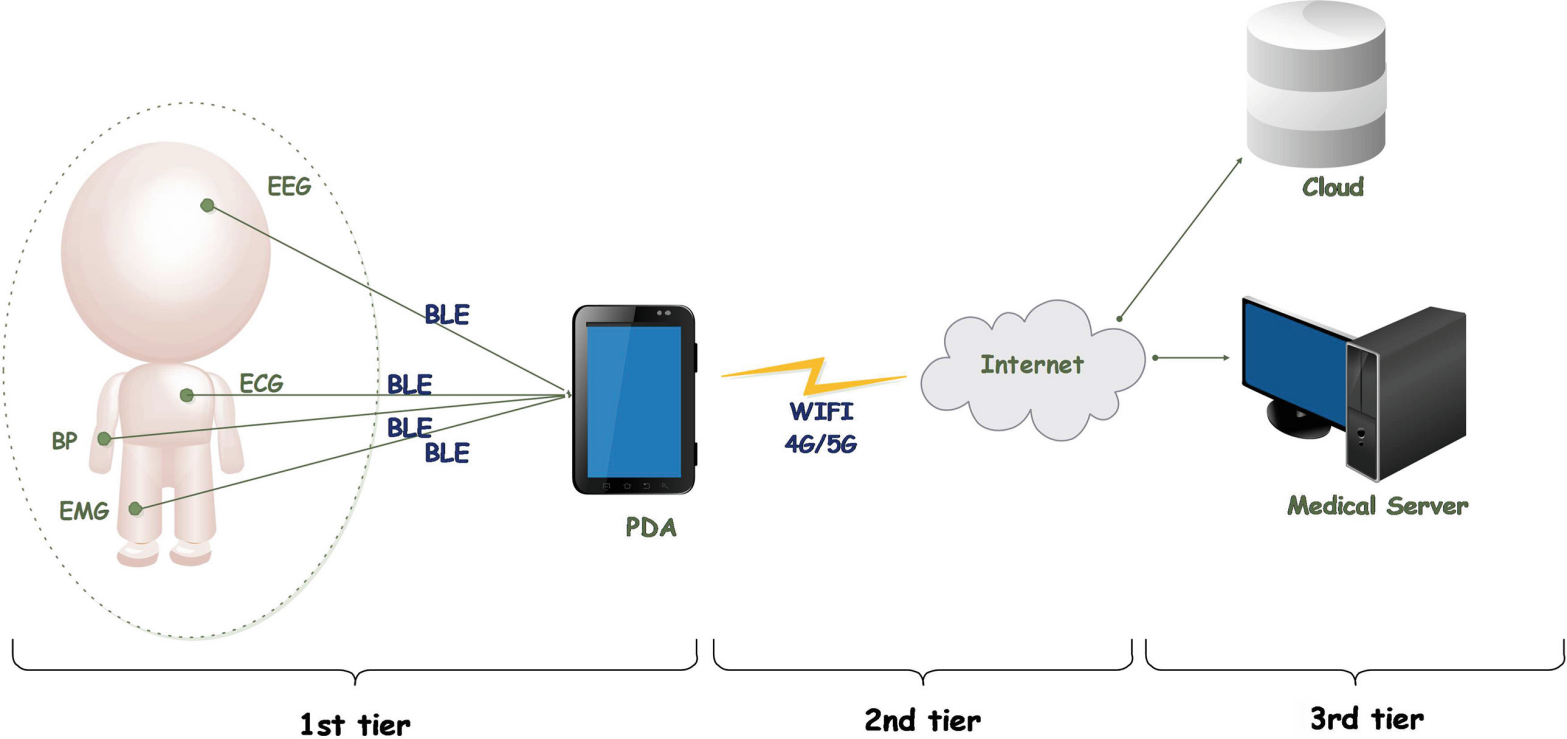
Remote healthcare monitoring system integrating BLE communication interfaces.

Generally, RHMS can be structured into three or four tiers [2]. In the following, we highlight the basic elements related to RHMS that are based on the three tiers architecture. The first tier involves the wearable sensors attached to the human body and the gateway or the coordinator. In this paper, the selected communication technology between the sensors and the PDA is BLE. Therefore the first tier which is the WBAN is composed by wearable sensors that sense the body vital signs (e.g. body temperature, heart rate, ECG, etc.) through the sensor nodes and sent them to the smartphone via BLE communication interfaces. The second tier that includes the networking infrastructure provides the connectivity between (1) the PDA and the remote medical servers, (2) between the PDA and the cloud. The third tier includes the medical web servers for data visualisation and the cloud (private: for security issues) for the data storage and processing. Figure 1 illustrates the RHMS three tiers architecture incorporating BLE communication interfaces.

### Related Works About RHMS Using BLE

Several communication technologies could be used in the first tier (as we mentioned in the introduction). In this section, we will focus only on the RHMS related works that are based on the BLE protocol for data gathering. We classified the studied contributions into two categories. The first category corresponds to RHMS that control or sense one physiological patient data. The second category resembles the RHMS using multiple sensors to sense several physiological data.

**Mono-Sensing RHMS.** In this subsection, we will analyze related works about RHMS that sense and collect physiological information from a single sensor.

***- Wearable Noncontact Armband for Mobile ECG Monitoring System:*** Rachim and all. [16] proposed to implement a mobile ECG monitoring system using a wearable noncontact armband ECG signal. The proposed system solved the problem of the previous healthcare heart devices which do not provide patient’s information such as heartbeat, heart disease, and heart conditions. The proposed system consists of capacitive-coupled electrodes fixed in an armband which is more convenient (smaller) than systems with ECG sensors strapped to the chest. The capacitive-coupled electrodes can sense bio-signals through the clothes. According to the experimental results carried by the authors, the developed system can still function with different clothing thicknesses between the sensors and the skin and when the user carries out various daily living activities. Furthermore, the authors developed an Android application showing in real-time the evolution of the ECG signal on a graph and analyze the sensed data for first notifications making.

***- Wireless Ring-Type Pulse Oximeter with Multi-detectors:*** The authors of [17] conceived and developed a wireless finger ring-type pulse oximeter with multi-detectors to monitor the blood oxygen saturation (SpO2). The developed system contains (1) three optical probes to provide the light source and receive the penetrated light that passes through the human tissue; (2) a wireless data acquisition module containing a microprocessor (MSP430), a LED driving circuit, PD amplifier circuits and a wireless transmission unit. PD amplifier circuits amplify and filter the received penetrated light. Therefore, the digitized penetrated light signal will be sent to the host system (that receive, display, store and analyze the penetrated light signal) using a wireless transmission unit including a Bluetooth v2.0 module and a printed circuit board (PCB) antenna. The monitoring program built in the host system will.

***- Wireless Scale Based on the Bluetooth 4.0 Low-Energy:*** Huang and all. [18] realized a low power and smart Bluetooth scale with a sensor chip CC2540 monitored through a mobile application. The weight sensors produce deformation and change their shapes when a patient ascend on the scale this lead to a variation of the driven voltage. Therefore, these signals are amplified and transmitted to an A/D converter.

***- Muscule Activity Monitoring:*** Several systems integrating EMG sensors to measure the electrical muscle activity and a BLE communication module to transmit gathered data to a mobile phone. In this context, authors in [19] designed a fabric stretch sensor embedded system for muscle activity monitoring. The strain sensor resistance varies sensitively with body movements. The designed system includes an application that shows muscle activity data and highlights the features such as muscle movement distribution. To avoid sports injuries during exercise, authors in [20] designed an EMG patch to supervise the muscle fatigue conditions during isotonic contraction. The developed system deploys two electrodes to measure the sEMG signal. A microcontroller unit in the EMG patch is used to measure in real-time the median frequency of an EMG signal. When the muscle is tired, the median frequency will shift to a low value. The sensed values are sent via BLE to a mobile phone running an APP that displays the muscle fatigue levels and the user riding information.

***- Brain Activity Monitoring:*** Sullivan and all. [21] proposed a brain activity monitoring system using the non-invasive electroencephalogram EEG sensor that measures the neural electrical activity of the brain from the scalp surface. The developed EEG monitoring system assisted by deep learning mechanism provides information about neonatal brain health to help clinicians in neonatal EEG abnormalities diagnosing. The proposed system uses a low-cost -low-power EEG acquisition system including BLE interface for communication. Besides that, the authors developed an Android app visualizing single-channel EEG and the neonatal seizure presence. A deep convolutional neural network and an algorithm for EEG sonification used to perceive EEG morphology changes.

***- Breath Rate Monitoring:*** Authors in [22] developed a new system to supervise in real-time the respiratory signal. The developed system includes three parts: smart belts, a display unit, and an online storage unit. A textile-based pressure fabric attached to a belt converting the stomach movement into an electrical signal that is transmitted via BLE to a remote station where it is displayed in real-time and uploaded to an online repository for future analysis. The authors tested the performance of the system when individuals performed activities like talking and walking. In [23] authors developed a system detecting sleep disorders such as respiratory flow repetitive cessations during sleep using a magnetometer sensor placed onto the body detecting millimetre night-time breathing movements by measuring the change in the magnetic vectors. The developed system includes a noninvasive wearable sensor, a wireless BLE module and a low-power microcontroller.

Table 1 gives a summary of all mono-sensing RHMS based BLE system.

**Table 1.** Recapitulative table related to monosensing RHMS based BLE.

Ref and date	Sensors	Placement	Applications
[16] 2016	ECG	Arm	Wearable Noncontact Armband for Mobile ECG Monitoring System
[17] 2014	SpO2	Finger	Oxygenated hemoglobin in the blood
[18] 2015	Weight	Outside the body	Low power and smart Bluetooth scale for weight monitoring.
[19] 2017	Strain	Fabric stretch sensor attached to the clothes	-Muscle activaty Monitoring and body motion recognition
[20] 2019	EMG	Lower leg, the gastrocnemius muscle	Real time monitoring of muscle attached to the clothes
[21] 2018	EEG	Scalp	Brain activity Monitoring
[22] 2018	PPG	Stomach movement	Respiratory rate monitoring

**Multi-sensing RHMS.** This subsection analyzes related works about RHMS that sense and collect physiological informations from more than one physioligical sensor.

***- Cardiovascular RHMS Using Multi-sensing:*** Authors in [30] designed an epidermal patch, which is called Chem-Phys that offers simultaneous real-time monitoring of a biochemical (lactate) and an electrophysiological signal (electrocardiogram) for fitness monitoring. Besides that, for monitoring the cardiovascular disease authors in [31] designed wearable devices such as ECG and heart rate that can be integrated into clothes or attached directly to the human body. In addition, Li Jinming and all. [26] proposed a multi-parameter cardiac remote monitoring system based on ECG, pulse rate and heart sound sensed data. This system is composed of a multi-channel physiological parameter acquisition unit, an Android terminal, a cloud server and a Bluetooth Low Energy BLE protocol for data communication. Besides that, [29] introduced heart-monitoring system evaluating the heart conditions based on sensed data from several wearable devices such as heart rate, blood pressure, body and skin temperature. The sensed patient information are transmitted to a smartphone (running an Android application) by the low energy protocol BLE and then will be visualized on the Web application. According the authors, evaluation of the developed monitoring system under expert’s supervision for 40 individuals (aged between 18 and 66 years) showed that the proposed system is convenient and reliable generating warning messages to the doctor and patient under critical circumstances.

***- Diabete Chronic Condition Monitoring Using Multi-sensing:*** Muhammad Syafrudin and all. [27] developed a healthcare monitoring system wich utilizing BLE based sensors to control the personal vital signs data such as heart rate, blood glucose, and blood pressure to support diabetic patients to manage individually their chronic condition. The BLE used in this system to transmit patient’s health information from sensors to the smartphone, while to manage the sensor data by utilizing the real-time data processing, which used the Apache Kafka as a platform and MongoDB as a database for storing the patient’s informations.

***- A Wearable Human Healthcare Monitoring System:*** proposed by [19] supervising personal’s information such as body temperature, heart rate, and blood oxygen saturation. The sensing node is wearable, miniaturized and based on the microchip CC2538 and Contiki OS. The sensed data are transmitted via a BLE communication interface to a mobile application. These data are stored on cloud server using the MQTT protocol and analyzed via the data mining approaches to diagnose user’s health status. In addition, authors in [28] designed a low-cost healthcare monitoring IoT-based system with the Fog layer sensing vital signs such as ECG, body temperature, and the respiration rate and contextual data (i.e. humidity and environment temperature).

***- Chronic Respiratory Monitoring Using Multi-sensing:*** James and all. [22] developed BLE-based RHMS to monitor chronic respiratory disease that sense multi-parameters. The system comprises a chest patch and a wristband. The chest patch sensors corresponds to ECG, PPG, motion and acoustic signal. The wristband sensors track ozone exposure, ambient relative humidity, ambient temperature, PPG and motion. The data from each sensor is transferring by BLE communication interface to the server for storage.

## BLE Communication Protocols

### Basic Concepts

Bluetooth v4.0 known as Bluetooth Low Energy is ideal for applications requiring sporadic or periodic transfer of small amounts of data. Thus, BLE is well suited for sensors, actuators and other small devices requiring low power consumption. BLE works well with high numbers of communication nodes with limited latency requirements, very low power consumption and short connection times and wake-up. BLE aims to provide the same communication range as classic Bluetooth while consuming less power. The most significant differences between BLE and Classic Bluetooth are (1) the BLE has a lower data rate, (2) BLE use just 40 channels (37 for data and 3 for advertising) instead of 79, (3) no support for audio and (4) simplified state machines. Both BLE and Classic Bluetooth, operates in the 2.4 GHz ISM (industrial scientific and medical) frequency band, precisely BLE frequency band range from 2.402 GHz to 2.480 GHz. To minimize the overlapping with other IEEE 802.11 channels, the three advertisement channels (37, 38, and 39) centered on 2,402 GHz, 2,426 GHz and 2,480 GHz. Advertising is a process required for devices to find each other. At the link layer BLE device, function as a state machine with four states: standby, scanning (master procedure), advertising (slave procedure), initiating and connection. Furthermore, BLE operates in piconets wit a star topology. The central node is the master and all other nodes in the piconet are slaves. BLE has an architecture client/server which the client can be the Master such as smartphone, gateway, etc and the server is the peripheral such as sensors.

The connection establishement occurs after sending connection request packet which handle connection parameters (connection interval, slave latency and supervision timeout).

***- Connection interval:*** corresponds to the time elapsed between two connection events. BLE devices are communicating only in connection events to save energy. So bigger interval between those events will save more energy but decrease data rate. No matter if device has data to send, it has to wait until next connection event. The interval can be set from 7.5 ms to 4 s.

***- Slave latency:*** is the number of connection events, that sensor node can skip to save energy without the risk of disconnected.

***- Connection supervision timeout:*** specifies the maximum time between two valid data received before a connection is lost.

The master coordinates the MAC using a TDMA scheme, determines the instants in which slaves are required to listen, and provides them with the map of data channels to be used.

### BLE Protocols Stack

BLE protocol stack involves two main elements: the controller and the Host. The Controller includes the physical layer and the link layer. Both implemented on a single chip with an integrated radio interface. The Host runs on an application processor. It covers five upper layer functionalities (the Logical Link Control and Adaptation Protocol (L2CAP), the Attribute Protocol (ATT), the Generic Attribute Profile (GATT), the Security Manager Protocol (SMP) and the Generic Access Profile (GAP). The standardized Host Controller Interface (HCI) provides the exchange between the Host and the Controller. An application layer can be developed on the top of the Host.

***- L2CAP:*** acts as an interface between the link layer and the Upper layer protocols (ATT, SMP and Link Layer control signaling). It multiplexes, segments, reassembles data packets and offers support quality of service management.

**Fig. 2.**
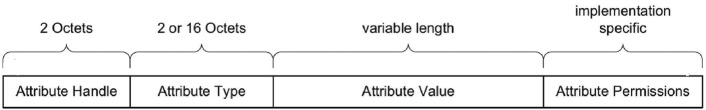
Attribute representation.

***- ATT:*** is a client/server stateless protocol based on attributes presented by a device. Each server holds data organized in the form of attribute managed by the GATT. Universally unique identifier (UUID), a set of permissions and a value identify each attribute (see Fig. 2).

***- SMP:*** offers security services for protecting the information exchange between two connected peers. It allows the generation and the exchange of security keys and it hide the public Bluetooth Address if required.

***- GATT:*** defines the GATT server and the GATT client and specifies the framework and operations for data transfer procedures over a BLE connection. A GATT client requests and receives data from a GATT server, whose makes the data available to the GATT client. Data is structured in sections called services that assembles related pieces of user data known as characteristics.

***- GAP:*** outlines rules and concepts to standardize the low-level operation of devices. It defines how devices perform control procedures such as device discovery, security establishment and connection...to guarantee interoperability between devices from different vendors.

## Reading Sensed Physiological Signs with BLE

The most difficult task confronted during mobile healthcare application development is sensed data reading. In this paper, we use BLE to get data from sensors and forward them to the PDA. In general, we develop an application for Android device (PDA: mobile phone) to get data from sensors. The complexity resides in the diversity of health devices (thermometer, heart-rate monitor, blood pressure monitor, scale ...), where each device has a specific profile, services, UUID, characteristics, and descriptors. Therefore, to read measured data from sensors, we need to develop a specific application for each profile. In this paper, we describe two health devices equipped with the BLE corresponding respectively to Health Thermometer and Blood Pressure Monitor.

### Example of Services and Characteristics of HDP

In order to standardize the way medical data is transmitted over BLE technology, the Bluetooth Special Interest Group (SIG) released in 2008 the Health Device Profile (HDP) that utilizes the IEEE 11073-20601 Data Exchange Protocol as the transport content. Below the specification of the service and characteristic of the studied health devices which according to the SIG group.

***Thermometer:***

*(1) Health Thermometer Service:* (UUID = 0x1809; Definition: This service exposes temperature and other data from a Health Thermometer Sensor;

Type = org.bluetooth.service.health-thermometer),

*(2) Temperature Measurement Characteristic* (UUID = 0x2A1C; Definition: This characteristic is used to send a temperature measurement;

Type: org.bluetooth.characteristic.temperature measurement).

***SPO2:***

*(1) Health PulseOximeter Service:* (UUID = 0x1822; Definition: This Service exposes pulse oximetry data related to a non-invasive pulse oximetry sensor for consumer healthcare applications; Type = org.bluetooth.service.pulse-oximeter),

*(2) PLX Continuous-Measurement* (UUID = 0x2A5F; Definition: This characteristic is used to send a PulseOximetre measurement;

Type: type = org.bluetooth.characteristic.plx-continuous-measurement).

Developers can obtains the services and characteristics of any BLE health device from the main Bluetooth webpage [24].

### Steps for Reading Sensed Data on Mobile App

The development of a Mobile App able to read BLE sensed data can be resumed by the following steps:

***Step 1:*** Declaring permission on a manifest file is required to use BLE features and to perform BLE communication toward requesting or accepting connection or reading the different measurements.

***Step 2:*** Assigning UUID for service and characteristic as declared in the previous subsection.

***Step 3:*** Chekinging that BLE is supported on the PDA and enable it.

***Step 4:*** Turning On the Bluetooth and displaying dialogue box on the PDA that asking for permission from the user to turn on Bluetooth.

***Step 5:*** Scanning and visualizing nearby BLE health devices and their addresses on the PDA.

***Step 6:*** Connecting to a GATT server on health device to manage the connection and to send data using the connectGatt() method that requires as parameters(a Context object, autoConnect and a reference to a BluetoothGattCallback). The BluetoothGattCallback is used to deliver GATT client operations to the client (Mobile App on the PDA).

***Step 7:*** Discovering, reading and displaying GATT services and characteristics by controlling the device activity (DeviceControlActivity).

***Step 8:*** Reading Sensed Data(for example: temperature). Thus, we set notification or indication value on Temperature Measurement Characteristic then write to the descriptor to set the right value for characteristics. The updates from the health device on characteristics value will be posted on the next callback using onCharacteristicChanged.

Developers can get detailed description and example of codes from the main android(section Bluetoot connectivity) webpage [25].

**Fig. 3.**
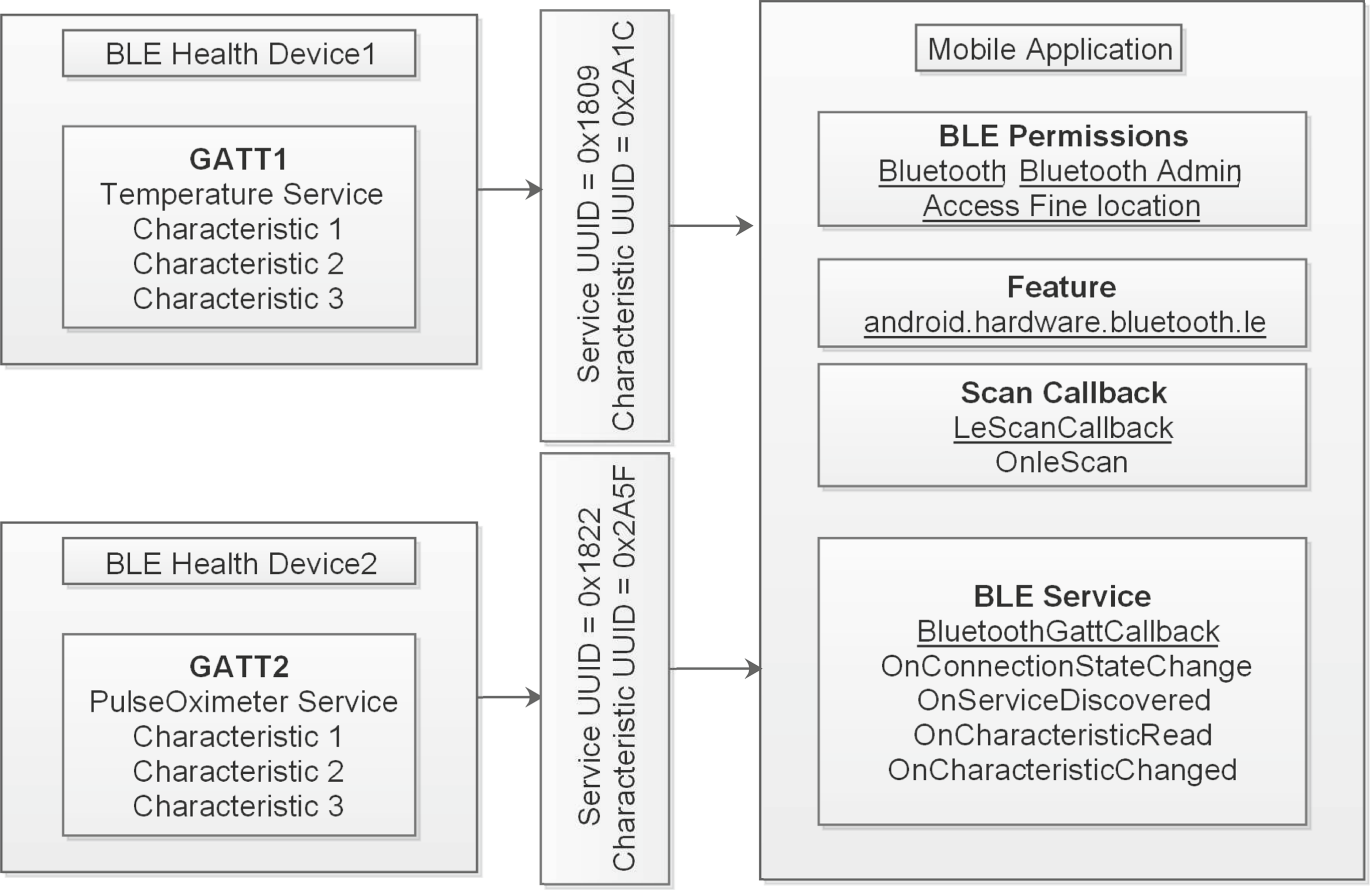
Required components for android BLE communication.

We recapitulate the different steps required for enabling a mobile App to get sensed data from health devices in Fig. 3.

## Conclusion

In this paper, we provided a detailed study related to RHMS based on BLE communication. We notice that several RHMS based BLE was developed and tested for different physiological signs, accordingly, in this paper, we studied and highlighted different mono and multi-sensing RHMS using BLE communication interfaces for physiological signs sensing. Besides that, we overviewed BLE communication in a comprehensive way to help readers and mobile app developers to understand the basic concepts of BLE communication. Furthermore, we detailed the different steps required for enabling sensor-side control and BLE communication on an android system as the mobile platform for the health sensor’s client application. We limited our investigation to sensed data reading. In an extended version of this work, we will provide a whole prototype that could be tested beyond the development environment and providing several vital signs monitoring (remote control, data visualization graphically) and decision making through the processing of sensed vital signs.
